# Trends in the recording of anxiety in UK primary care: a multi-method approach

**DOI:** 10.1007/s00127-021-02131-8

**Published:** 2021-07-01

**Authors:** Charlotte Archer, Katrina Turner, David Kessler, Becky Mars, Nicola Wiles

**Affiliations:** 1 Bristol Medical School, Centre for Academic Mental Health, University of Bristol, Population Health Sciences, Bristol, BS8 2BN UK; 2 NIHR Bristol Biomedical Research Centre, Bristol, UK; 3 The National Institute for Health Research, Applied Research Collaboration West (NIHR ARC West), University Hospitals Bristol NHS Foundation Trust, Bristol, UK

**Keywords:** General practice, Primary care, Trends, Anxiety disorders, Mental health, Multi-methods

## Abstract

**Purpose:**

Anxiety disorders are common. Between 1998 and 2008, in the UK, GP recording of anxiety symptoms increased, but the recording of anxiety disorders decreased. We do not know whether such trends have continued. This study examined recent trends in the recording of anxiety and explored factors that may influence GPs’ coding of anxiety.

**Methods:**

We used data from adults (*n* = 2,569,153) registered with UK general practices (*n* = 176) that contributed to the Clinical Practice Research Datalink between 2003 and 2018. Incidence rates and 95% confidence intervals were calculated for recorded anxiety symptoms and diagnoses and were stratified by age and gender. Joinpoint regression was used to estimate the years trends changed. In addition, in-depth interviews were conducted with 15 GPs to explore their views and management of anxiety. Interviews were audio-recorded, transcribed verbatim and analysed thematically.

**Results:**

The incidence of anxiety symptoms rose from 6.2/1000 person-years at risk (PYAR) in 2003 to 14.7/1000 PYAR in 2018. Between 2003 and 2008, the incidence of anxiety diagnoses fell from 13.2 to 10.1/1000 PYAR; markedly increasing between 2013 and 2018 to 15.3/1000 PYAR. GPs mentioned that they preferred using symptom codes to diagnostic codes to avoid assigning potentially stigmatising or unhelpful labels, and commented on a rise in anxiety in recent years, especially in young adults.

**Conclusion:**

Recent increases in the recording of both anxiety diagnoses and symptoms may reflect increased presentation to primary care, especially in young adults. There is a clear need to understand the reasons for this, and this knowledge may be critical in the prevention and treatment of anxiety.

**Supplementary Information:**

The online version contains supplementary material available at 10.1007/s00127-021-02131-8.

## Introduction

Anxiety disorders are common. Within the European Union, approximately 38.2% of the population experience a mental disorder each year, of which anxiety is the most prevalent (14%) [1]. In UK general practice, the prevalence (7.2%) and incidence (9.7 per 1000 person-years) of anxiety disorders is high [2]. There is evidence that how GPs record presentations of anxiety have changed over time. Between 1998 and 2008, GP recording of anxiety symptoms increased (3.9/1000 PYAR to 5.8/1000 PYAR), whereas recording of anxiety disorders decreased (7.9/1000 PYAR to 4.9/1000 PYAR) [3]. Others have also reported an increase in symptom codes for anxiety and depression combined [4] and a recent increase in anxiety codes (1998–2018)[5]. However, Slee et al. [5] focused on generalised anxiety disorder (GAD) and combined symptom and diagnosis codes. Hence no data are available on whether GP recording of presentations of anxiety in UK primary care has changed in recent years.

Several factors may have impacted incidence rates and changed how GPs record anxiety: the 2006 depression Quality Outcomes Framework (QOF) [6]; the Improving Access to Psychological Therapies (IAPT) service introduced in 2007/2008; the 2008 recession; and the 2011 NICE anxiety guidelines [7]. The latter recommended that “recognition and communication of the diagnosis of GAD should occur as early as possible to help people understand the disorder and start effective treatment promptly” (page 7) [7].

The fall in recorded anxiety diagnoses [3] may be due to a reluctance by GPs to formally label patients with an anxiety disorder, or a preference for using broad symptom codes rather than distinguishing between the subtypes of anxiety [8, 9]. However, the reasons are unclear as few UK studies have explored GP views on the diagnosis of anxiety.

The aim of this study was to examine trends in the recording of anxiety in UK primary care between 2003 and 2018 using Clinical Practice Research Datalink (CPRD) data and to investigate whether there were differences according to age and gender. In addition, qualitative interviews with GPs were conducted alongside this study to provide insight into possible reasons for the trends observed.

## Methods

### Data source

The CPRD GOLD is a large database of anonymised UK primary care electronic records and is considered broadly representative of the UK population regarding gender and age [10, 11]. Contributing practices use Vision practice management software [12]. For each registered patient, the record contains information including consultation dates and prescriptions. At the time of conducting this research, practices sampled used the READ code system [13] to record symptoms or diagnoses. These codes may be recorded by either GPs or practice nurses.

### Study population

We used data from adults aged 18 years and over, registered at a CPRD practice between 1st January 2003 and 31st December 2018. CPRD assigns quality metrics: ‘acceptable’ patient records are those with valid registration and demographic information, with no breaks in the record; and ‘up-to-standard’ practices are those with continuous accurate recording of data, including patient deaths or transfers out of practice [11]. Patient records had to be ‘acceptable’ and from practices that were ‘up-to-standard’ for at least one year prior to the study entry date. Practices had to have contributed data for the entire study period. Patients with a recorded anxiety code had to have been registered with CPRD for one year before the first recorded code to ensure high-quality assessment of incident cases.

### Codes for anxiety

Those with a recorded anxiety diagnosis and/or recorded symptoms of anxiety were identified using codes compiled from the NHS Anxiety READ Codes (Version 3, April 2018), and cross-checked with code lists from previous epidemiological research [3, 4] (Supplement 1). This included using code lists created in these previous studies to delineate between anxiety symptoms and anxiety disorders for the present study [3, 4]. These lists were then reviewed by a GP, with no further codes added or excluded. All authors agreed on the final list. Codes for obsessive–compulsive disorder and post-traumatic stress disorder were excluded as these disorders are no longer included as ‘anxiety disorders’ in the Diagnostic and Statistical Manual (DSM-5) [3, 4, 14, 15]. We did not include phobias as our focus was on generalised anxiety disorder and related disorders, rather than specific phobias, which would require a different treatment approach.

### Statistical analysis

Data analysis was conducted using Stata version 15.1 [16].

Incident use of codes in each calendar year was examined in terms of those with a new episode defined by: (1) any anxiety code (symptom or diagnosis); (2) a diagnosis code; and (3) a symptom code. A new episode was defined as a recorded symptom or diagnosis of anxiety in that year, with no prior recorded code of that category recorded in the previous 12 months. Patients could have more than one new episode within the study period provided that there was a minimum of 12 months between episodes.

Annual incidence rates were calculated by dividing the number of incident cases by the total person-years at risk (PYAR) for each calendar year, and are presented per 1000 PYAR. Data were stratified by age (< 25, 25–34, 35–44, 45–54, 55–64, 65–74, 75–84, ≥ 85 years) and gender. Estimates of 95% confidence intervals (95% CI) for these rates were calculated based on the Poisson distribution.

Univariable Poisson regression models were used to examine the association between year of recording, age, gender, and incidence of anxiety symptoms/diagnoses. Incidence rate ratios (IRRs) and 95% CIs are reported. Multivariable Poisson regression models that included year, age and gender were used to examine the independent effects of such factors. Sensitivity analyses were conducted to account for any clustering within practices within the multivariable model. To formally test whether incidence varied over time according to age and gender, the multivariable Poisson regression model was repeated including an interaction between gender and year, and age and year.

Changes in trends over time were examined using joinpoint regression (version 4.7.0.0) (National Cancer Institute, 2020). By fitting a series of joined straight lines, the model selects the point(s) at which there is a significant increase/decrease in the rate (joinpoints) thus identifying the years (with 95% CI) at which changes in trends occurred. The annual percentage change (APC), based on the slope of each line between joinpoints, was also calculated.

Additional analysis were undertaken grouping diagnosis codes based on ICD-10 classification [17] in terms of generalised anxiety (GAD), mixed anxiety and depression (MADD), or panic attacks or disorders (Panic). Annual incidence rates (and 95% CI) were calculated for each diagnostic group as described earlier.

### Qualitative interviews

#### Recruitment, sampling, and data collection

Practices in Bristol and the surrounding area were informed about the study by the Clinical Research Network. GPs willing to be interviewed completed a response form and emailed it to the research team. GPs were purposively sampled to achieve maximum variation in relation to gender, age and length of time working in primary care. GPs were also sampled based on practice deprivation decile, and socio-demographic characteristics of their patients.

GPs were interviewed by CA, having given informed consent to participate. A topic guide was used to ensure consistency across the interviews, with interviewees asked about causes of anxiety and the codes they use. For full details see Archer et al. [18].

#### Data analysis

Data collection and analysis proceeded in parallel so that initial interviews informed later interviews. Data collection ended when data saturation was reached (no new themes identified). Interviews were audio-recorded, transcribed verbatim, anonymised, and checked for accuracy.

Data were analysed thematically [19]. CA and KT independently coded a sub-set of transcripts, and then compared and discussed their coding and interpretation of the data. A preliminary coding framework was developed, which was revised as new codes were identified in later transcripts. All transcripts were coded electronically in NVivo (version 12) [20]. Data under specific codes were then retrieved, and read and compared to identify key themes and deviant cases. Interviews were fully analysed before analysis of the CPRD data.

## Results

### CPRD data

#### Sample characteristics

The dataset included 176 practices with 2,569,153 eligible patients registered across the 16-year period (2003–2018), and 17.6 million person-years of follow-up (PYFU). Over the study, there were 264,127 incident anxiety codes (any anxiety code) recorded; 216,126 new episodes of anxiety diagnoses; and 197,217 new episodes of anxiety symptoms.

#### GP use of anxiety codes

GPs used a large number of READ codes (Table 1). The most frequently used diagnostic codes were ‘anxiety states’, ‘anxiety with depression’, and ‘panic attack’, totalling 82.6% (*n* = 178,488/216,126) anxiety diagnosis episodes. ICD-10 diagnostic codes were used infrequently, with ‘generalised anxiety disorder’ and ‘mixed anxiety and depressive disorder’ each representing less than 2% (*n* = 3,482/216,126; *n* = 3,735/216,126) of diagnostic codes. When diagnostic codes were grouped, codes relating to GAD accounted for more than half of diagnosis codes used by GPs, with a further 31% attributed to the MADD category. When recording anxiety symptoms, ‘anxiousness symptom’, ‘anxiousness’, and ‘worried’, were used in the majority (*n* = 192,243; 97.5%) of anxiety symptom episodes.

**Table 1:** Frequency of Read codes used by GPs to record anxiety diagnoses and symptoms

	Code	Total	
		Freq	%
Diagnosis codes	Anxiety states	93,989	43.5
	Anxiety with depression	61,831	28.6
	Panic attack	22,668	10.5
	Anxiety state NOS	7,301	3.4
	Panic disorder	5,740	2.7
	[X] Mixed anxiety and depressive disorder	3,735	1.7
	Generalised anxiety disorder	3,482	1.6
	Chronic anxiety	3,125	1.5
	Anxiety state unspecified	3,095	1.4
	Agoraphobia with panic attacks	1,879	0.9
	[X] Anxiety disorder, unspecified	1,549	0.7
	[X] Mild anxiety depression	1,091	0.5
	[X] Anxiety NOS	991	0.5
	[X] Other anxiety disorders	984	0.5
	[X] Generalised anxiety disorders	928	0.4
	Recurrent anxiety	703	0.3
	[X] Agoraphobia	630	0.3
	[X] Panic attack	528	0.2
	[X] Panic disorder (episodic paroxysmal anxiety]	430	0.2
	[X] Social phobias	410	0.2
	Social phobic disorders	226	0.1
	[X] Persistent anxiety depression	204	0.1
	Agoraphobia without mention of panic attack	153	0.1
	[X] Anxiety state	140	0.1
	[X] Anxiety neurosis	135	0.1
	[X] Panic state	83	0.0
	[X] Panic disorder with agoraphobia	49	0.0
	[X] Other mixed anxiety disorders	29	0.0
	[X] Other specified anxiety disorders	12	0.0
	[X] Agoraphobia without history of panic disorder	3	0.0
	[X] Social neurosis	3	0.0
	Total	216,126	100
Diagnosis codes—sub-type group	Generalised anxiety (GAD)	112,898	52.2
	Mixed anxiety and depression (MADD)	66,861	30.9
	Panic attack or disorder (PANIC)	29,449	13.6
	Other anxiety codes	6,918	3.2
	Total	216,126	100
Symptom codes	Anxiousness symptom	104,278	52.9
	Anxiousness	69,775	35.4
	Worried	18,220	9.2
	Anxious	2,532	1.3
	Nerves	958	0.5
	O/E—anxious	923	0.5
	Tension—nervous	448	0.2
	O/E panic attack	64	0.0
	Nervous—nervousness	19	0.0
	Total	197,217	100

#### Trends in coding over time

**Fig. 1:**
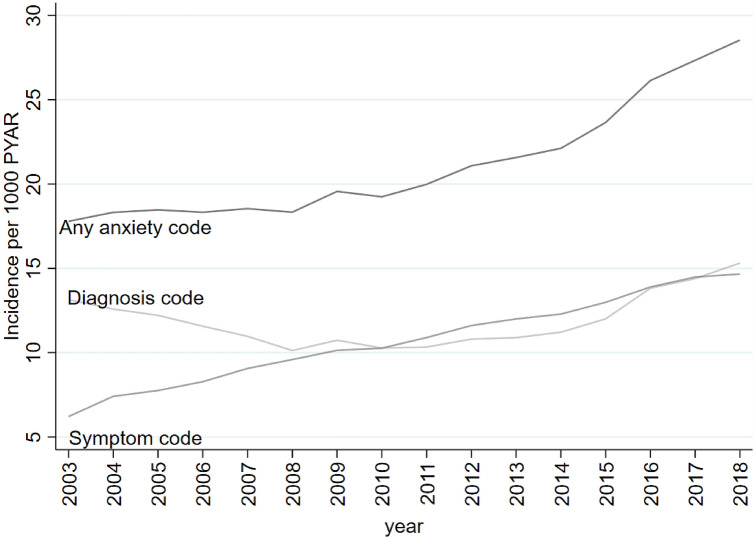
Trends in the incidence of GP recorded anxiety (any code, diagnosis, and symptom codes) between 2003 and 2018

The incidence of any anxiety code rose from 17.8/1000 PYAR in 2003 to 28.5/1000 PYAR in 2018. Between 2003 and 2008, the incidence of anxiety diagnoses fell from 13.2/1000 PYAR to 10. 1/1000 PYAR; after which incidence remained fairly constant, before increasing in recent years (Table 2 and Fig. 1). The incidence of anxiety symptoms more than doubled over the entire study period rising from 6.2/1000 PYAR in 2003 to 14.7/1000 PYAR in 2018 (Table 2 and Fig. 1).

**Table 2:** Incidence (crude) rates per 1000 PYAR for GP recorded anxiety—any anxiety code, anxiety diagnoses and anxiety symptoms—between 2003 and 2018

Variable		Any anxiety code				Diagnoses				Symptoms			
		No. of events	PYAR	Incidence (1000 PYAR)	(95% CI)	No. of events	PYAR	Incidence (1000 PYAR)	(95% CI)	No. of events	PYAR	Incidence (1000 PYAR)	(95% CI)
Year	2003	19,653	1,104,840	17.8	(17.5–18.0)	14,560	1,107,325	13.2	(12.9–13.4)	6905	1,111,271	6.2	(6.1–6.4)
	2004	20,174	1,101,094	18.3	(18.1–18.6)	13,957	1,108,836	12.6	(12.4–12.8)	8295	1,118,817	7.4	(7.3–7.6)
	2005	20,139	1,090,525	18.5	(18.2–18.7)	13,476	1,103,323	12.2	(12.0–12.4)	8668	1,117,151	7.8	(7.6–7.9)
	2006	19,969	1,089,605	18.3	(18.1–18.6)	12,808	1,107,044	11.6	(11.4–11.8)	9301	1,123,201	8.3	(8.1–8.5)
	2007	20,165	1,087,647	18.5	(18.3–18.8)	12,172	1,109,495	11.0	(10.8–11.2)	10,215	1,126,579	9.1	(8.9–9.2)
	2008	20,009	1,091,521	18.3	(18.1–18.6)	11,324	1,117,947	10.1	(9.9–10.3)	10,884	1,134,517	9.6	(9.4–9.8)
	2009	21,323	1,089,956	19.6	(19.3–19.8)	12,036	1,120,938	10.7	(10.6–10.9)	11,525	1,136,496	10.1	(1.0–10.3)
	2010	21,006	1,091,637	19.2	(19.0–19.5)	11,582	1,126,857	10.3	(10.1–10.5)	11,723	1,141,488	10.3	(10.1–10.5)
	2011	21,808	1,091,322	20.0	(19.7–20.3)	11,685	1,130,669	10.3	(10.2–10.5)	12,465	1,143,857	11.0	(10.7–11.1)
	2012	23,114	1,096,434	21.1	(20.8–21.3)	12,318	1,140,092	10.8	(10.6–11.0)	13,372	1,151,609	11.6	(11.4–11.8)
	2013	23,645	1,096,102	21.6	(21.3–21.9)	12,456	1,143,493	10.9	(10.7–11.1)	13,846	1,153,634	12.0	(11.8–12.2)
	2014	24,320	1,099,656	22.1	(21.8–22.4)	12,910	1,150,993	11.2	(11.0–11.4)	14,250	1,159,472	12.3	(12.1–12.5)
	2015	26,088	1,103,179	23.7	(23.4–23.9)	13,907	1,158,402	12.0	(11.8–12.2)	15,137	1,165,447	13.0	(12.8–13.2)
	2016	28,952	1,107,757	26.1	(25.8–26.4)	16,137	1,167,097	13.8	(13.6–14.0)	16,305	1,173,397	14.0	(13.7–14.1)
	2017	30,252	1,106,657	27.3	(27.0–27.7)	16,835	1,169,467	14.4	(14.2–14.6)	17,031	1,175,674	14.5	(14.3–14.7)
	2018	31,582	1,106,771	28.5	(28.2–28.9)	17,963	1,173,081	15.3	(15.1–15.5)	17,295	1,179,517	14.7	(14.5–14.9)

The best-fitting joinpoint model for any anxiety codes included one joinpoint at 2011 [with 95% confidence that the joinpoint was between 2009 and 2014 (95% CI 2009–2014)], after which incidence substantially increased (Supplement 2). For diagnosis codes, the best fitting model included two joinpoints: one in 2008 (95% CI 2006–2011), after which incidence remained fairly constant, and one in 2013 (95% CI 2011–2016), after which incidence substantially increased (Supplement 4). For symptom codes, the best fitting model had one joinpoint at 2007 (95% CI 2005–2009), after which incidence continued to increase, but at a slower rate compared with earlier years (Supplement 5).

After adjusting for age and gender, the IRR for any anxiety code was 1.65 (95% CI 1.63–1.68) when comparing 2018 with 2003 (Supplement 5). For symptom codes, after adjusting for age and gender, incidence more than doubled [IRR 2.41 (95% CI 2.34–2.48)] when comparing 2018 with 2003 (Supplement 7).

Recorded incidence of anxiety in females was nearly twice that of males (Supplement 5–7). This was consistent across any anxiety code, anxiety diagnoses and anxiety symptoms [adjusted IRR: females compared with males: any anxiety code IRR 2.13 (95% CI 2.11–2.14); diagnosis codes IRR 2.07 (95% CI 2.05–2.09); symptom codes IRR 2.12 (95% CI 2.10–2.14)] (Supplement 5–7).

Recorded incidence of anxiety (any code) decreased with age, with incidence for those aged ≥ 85 being just over half (IRR: 0.58 (95% CI: 0.57–0.60)) that of those aged < 25 (Supplement 5). A similar pattern was found for recorded incidence for anxiety diagnoses (Supplement 7), with the incidence for those aged ≥ 85 being approximately half [IRR: 0.48 (95% CI: 0.46–0.50)] that of those aged < 25, and for anxiety symptoms (Supplement 6), with a 30% reduction in the incidence of anxiety for the oldest age group compared with the youngest age group [IRR: 0.67 (95% CI 0.65–0.69)].

Findings from sensitivity analyses examining the potential impact of clustering within GP practices were consistent with the results from models that did not allow for clustering (data not shown). IRRs were the same, although confidence intervals were slightly wider.

#### Trends in coding over time by gender and age

Whilst the recorded incidence of anxiety was more common in females, the overall pattern of trends over time (any anxiety code, diagnoses and symptoms) were similar for males and females (Supplement 8–10). There was no evidence of an interaction between year and gender for any anxiety code (*p* value for interaction = 0.38). Whilst there was evidence of an interaction between year and gender for diagnosis codes (*p* < 0.001) and (weakly for) symptom codes (*p* = 0.053), the differences were small and unlikely to be clinically meaningful.

**Fig. 2:**
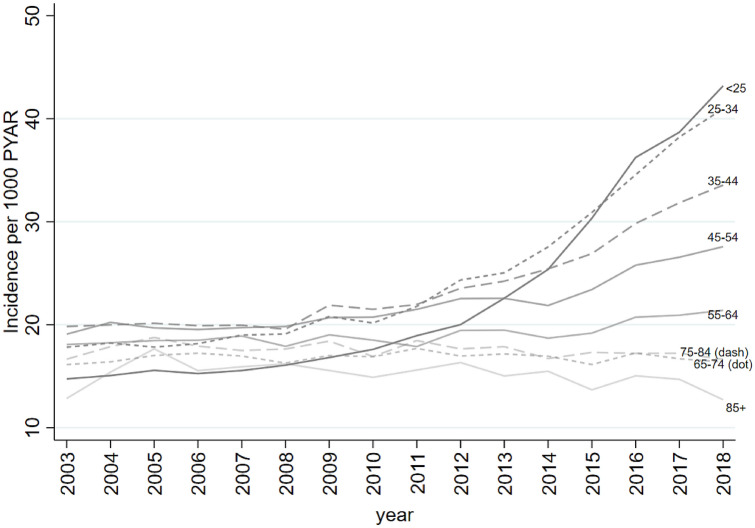
Incidence of GP recorded anxiety (any anxiety code) per 1000 PYAR, by age

When stratified by age, recorded incidence increased substantially in younger age groups in later years of the study (Fig. 2 and Supplement 11 and 12). There was strong evidence of an interaction between year and age for all models (any anxiety code, diagnosis, and symptoms: *p* value for interaction < 0.001).

There was a marked increase in the recorded incidence of anxiety diagnosis between 2013 and2018 in the two youngest age bands, increasing from 11.8/1000 PYAR to 24.4/1000 PYAR for under 25 s and from 13.1/1000 PYAR to 22.7/1000 PYAR for those aged 25–34. Incidence of anxiety diagnosis fell between 2003 and 2018 in the oldest age groups, decreasing from 10. 5/1000 PYAR to 8.1/1000 PYAR for those aged 75–84, and from 8.4/1000 PYAR to 6.1/1000 PYAR for those aged ≥ 85 (Supplement 11).

There was a marked increase in the recorded incidence of anxiety symptoms over the duration of the study for the two youngest age bands, increasing from 4.6/1000 PYAR to 22.2/1000 PYAR for under 25 s and from 5.7/1000 PYAR to 21.2/1000 PYAR for those aged 25–34. In contrast, whilst the incidence of anxiety symptoms increased over the first half of the study period for the oldest age groups (65–74, 75–84, and ≥ 85 years), incidence then decreased in the second half (Supplement 12).

#### Trends in coding over time of diagnosis subtypes

**Fig. 3:**
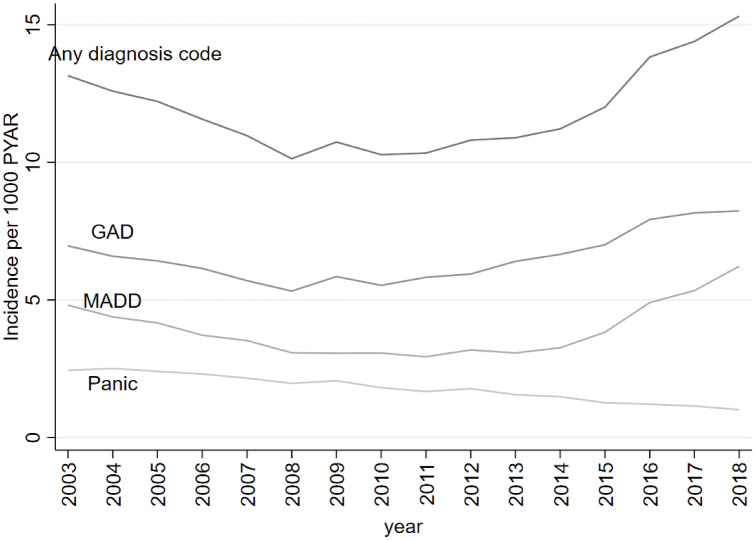
Trends in the incidence of GP recorded anxiety—any diagnosis code, generalised anxiety (GAD), mixed anxiety and depression (MADD), and panic attack/disorder (Panic)—between 2003 and 2018

Trends over time in the diagnosis subtype groups were also examined (Fig. 3 and Supplement 13). Between 2003 and 2008, the incidence of GAD fell from 7.0 to 5.3/1000 PYAR; increasing over subsequent years to 8.2/1000 PYAR in 2018 (Fig. 3 and Supplement 13). The incidence of MADD gradually decreased from 4.8/1000 PYAR in 2003 to 2.9/1000 PYAR in 2011; and then increased to 6.2/1000 PYAR in 2018 (Fig. 3 and Supplement 13). Between 2003 and 2018, the incidence of Panic gradually declined from 2.4/1000 PYAR to 1.0/1000 PYAR (Fig. 3 and Supplement 13).

### Qualitative interviews

Fifteen GPs from six GP practices were interviewed between September 2018 and March 2019. The mean age was 44.9 years (SD 7.7) and eight interviewees were female (53.3%). Those interviewed had been consulting in general practice between 4 and 27 years. The analysis focused on identifying factors that may explain the trends seen in GP coding of anxiety. Possible reasons for these trends are detailed below.

#### Recent increases in anxiety

GPs commented that the number of patients presenting with anxiety had increased over time, and this had increased GP awareness of anxiety. They suggested that increasing internet use for shopping, working, and interacting meant “people are becoming more isolated…they’re not having to go out…[and] interact with people as much” (GP 15) and lacked ‘real-life’ social support. GPs stated that social media may skew perceptions of what real life is like and made it much easier to make comparisons with others’ lives, and that this could be a factor in the increase in anxiety.

“Social media…this perception that everyone should have this perfect life, perfect looks, perfect body, perfect house, perfect holidays...the reality is that not everyone has…I think that’s what’s feeding an anxiety boom.” GP 1

GPs reported that they had seen a recent increase in patients aged “18–25” (GP 11) presenting with anxiety, notably in the past five years.

“I’ve been a GP for 20 years and the incidence of anxiety seems to be on the increase, especially in the last five years, especially in younger people.” GP 12

They suggested this could be driven by social media, and by “an awful lot more pressure, or perceived pressure…to either perform or to do things” (GP 12), such as “exam…social…work performance” (GP 7). GPs commented that this could be compounded by online gaming. They are living in a “virtual world [that] is not the real world…losing social and physical contact…it makes them anxious about going out and [having] social contact.” (GP 13).

GPs stated that they felt in recent years there had been increased awareness of anxiety in the media and by celebrities, with “greater recognition from the public of their symptoms, less stigma, and [therefore more likely to] seek help” (GP 1). They explained that this meant patients often knew they ‘had’ anxiety and would “specifically raise the question themselves” (GP 4). Therefore, there was potentially an expectation that the GP ‘had’ to “medicalise” (GP 11) their symptoms and give a label of anxiety.

“By the time it gets to us we’re probably over-pathologising it, because we’re seeing it so we’re kind of feeling we have to do something about it…it’s quite difficult just to say that’s normal, don’t worry about it.” GP 2

#### Coding choice and influences

GPs commented they that used codes such as “anxiety states” (GP 9) to cover a general sense of anxiety, rather than ICD-10 codes. GPs talked about progressing to other diagnostic codes when they had more information during follow-up consultations, such as “generalised anxiety…or…chronic anxiety if they’d had [past] episodes” (GP 10). However, some GPs talked about using codes interchangeably, with a tendency to select whichever anxiety code presented first on the list—“whatever comes up first, that’s a code for anxiety, that’ll do” (GP 2). When talking about anxiety and depression presenting co-morbidly, GPs reported a tendency to code for both conditions “under the umbrella of depression” (GP 11).

“It can be difficult if someone’s depressed and having panic attacks, and I think the majority I do put as depression, but if someone has predominantly anxiety then I will classify them often as depression with anxiety.” GP 12

GPs preferred to use symptoms codes rather than label patients with an anxiety disorder because they thought they could be stigmatising, or because patients “don’t want to be given a diagnosis” (GP 4). Several GPs stated that coding for anxiety was particularly unhelpful if “it makes it easier for them to assume the sick role…that they’re not getting better” (GP 13).

Some GPs mentioned the depression QOF as influencing the decision to code for a symptom rather than a disorder. Although they referred to depression rather than anxiety, there was a sense that the QOF had led to GPs being more cautious about using diagnostic codes across all mental health conditions.

“I think QOF…has skewed prevalence rates…because now if we write depression [rather than low mood] they chastise us if we haven’t done so much within a number of weeks… so I tend to be rather cautious about labels.” GP 6

#### Threshold for coding symptom versus diagnosis

GPs reported that severity and chronicity of symptoms were used to determine “when to change [the code] to [an] anxiety disorder, where they have chronic…anxiety, like long-standing” (GP 11), rather than using a symptom code. Some GPs suggested they would delay coding for a disorder until “six weeks, a month…if they’re still managing to work then I probably would delay the diagnosis longer” (GP 1).

Some GPs commented that there was an association between coding for an anxiety disorder and prescribing medication to treat it. That is, if a patient had reached a threshold for being prescribed medication, then they would have also reached the threshold for an anxiety diagnosis, rather than an anxiety symptom.

“If I was prescribing an SSRI for anxiety without depression, I would certainly make a formal diagnosis [with a diagnostic code] then.” GP 6

## Discussion

### Summary

The recorded incidence of anxiety symptoms increased between 2003 and 2018. In contrast, the recorded incidence of anxiety diagnoses decreased between 2003 and 2008, before markedly increasing between 2013 and 2018. When subdivided by diagnostic category, GAD and MADD showed a similar trend. However, the recorded incidence of Panic gradually declined across the 16-years.

Recorded incidence in females was nearly twice that of males—for any anxiety code, diagnosis codes, and symptom codes. Recorded incidence—any code, diagnosis, and symptoms—increased substantially in the later years of the study in younger age groups (18–34 years). There was also an increase in recorded incidence in recent years for 35–54-year-olds, although it was less marked. The recorded incidence for the older age groups (≥ 65 years) declined in later years.

GPs’ accounts suggested that GPs prefer to use symptom codes to diagnostic codes. Symptom codes were used for acute and mild anxiety, and diagnostic codes used for chronic and severe anxiety. This may, in part, explain the increase in the recorded incidence of anxiety symptoms over the study, and the decrease in anxiety diagnoses over the first five years. Hence, the trends observed may reflect changes in GP recording, rather than solely a change in incidence. However, GPs also commented on a recent rise in primary care presentation of anxiety and suggested a greater awareness of anxiety in society as a possible reason for this. This may explain the increase in the incidence of recorded diagnostic codes in later years. Additionally, the severity or chronicity of anxiety may have increased over recent years, and this could also be driving the increasing use of diagnostic codes. This increased incidence may also be due to better detection of anxiety by GPs and increasing acknowledgement of anxiety as a distinct disorder from depression. Cases that would have been previously coded as depression, may now being coded as anxiety. GPs commented on increasing anxiety in young adults. This is consistent with the increase in recorded incidence—of both diagnosis and symptoms—found in 18–34-year-olds in recent years in the CPRD data. GPs suggested this could be due to social media and pressure on young adults.

### Strengths and limitations

This study reports trends in a large sample that can be considered representative of the UK population, over a 16-year period. An extensive code list was used, compiled from national READ code terms, and cross-checked with code lists from previous epidemiological research [3]. It is therefore likely to capture most anxiety codes used by GPs, and prior research has validated such diagnoses recorded by GPs in primary care research databases [2]. A multi-method approach enabled the exploration of possible reasons for the trends observed. Interviews were analysed prior to analysis of the CPRD data, and therefore not influenced by knowledge of the quantitative findings. GPs were interviewed who varied in terms of age, gender, practice deprivation decile, and experience.

In terms of limitations, the quantitative sample is comprised of patients with a recorded anxiety code. It is likely that there are individuals with anxiety who have discussed symptoms with their GP, but have not had it coded within their record, or only recorded in free-text. Similarly, individuals whose anxiety is not detected, or where GPs have not coded it separately from depression or physical health conditions, will also not be included. Hence, the reported figures may be an underestimate of the incidence of anxiety, or the trends seen may be biased if GPs use of free-text recording has changed over time. In addition, trends have only been reported for those who consult for anxiety. No adjustment was made for the level of deprivation, however previous research in this area has indicated that adjustment for deprivation does not materially affect the reported trends in the coding of anxiety [3]. In terms of the qualitative data, the participating GPs were self-selecting. It is possible that those interviewed had more of an interest in anxiety than those who did not respond to the invitation.

### Comparisons with existing literature

Walters et al. [3] examined the incidence of anxiety in primary care between 1998 and 2008 and found an increase in recorded anxiety symptoms. Our findings extend this work and show that this trend has continued over the subsequent ten years. However, whilst Walters et al. [3] found a decrease in the incidence of anxiety disorders between 1998 and 2008, the present study found that incident anxiety diagnoses increased after 2008. When comparing incidence rates for the overlapping years (2003–2008), Walters et al. [3] found symptom rates rose from 3.9 to 5.8/1000 PYAR, whereas higher rates of 6.2–9.6/1000 PYAR were seen in this study. The higher rates may be due to the additional READ codes included in the present study, that accounted for 11% of recorded symptom codes. The decline in the incidence of GAD in this study to 2008 (5.3/1000 PYAR) is consistent with the recorded decrease in the incidence of GAD codes observed at the end of Walters et al. [3] study (4.9/1000 PYAR in 2008). Similarly, there was a trend of reduced incidence of Panic and MADD during the overlapping years in the two studies. Slee et al. [5] examined the incidence of GAD in primary care between 1998 and 2018 and found an increase in anxiety diagnoses and symptoms combined. Our findings of an increase in the incidence of any anxiety code between 2003 and 2018 are consistent with this.

John et al. [4] focused on GP coding for anxiety and depression between 2000 and 2009 and reported an increase in symptom codes, but a stable incidence of diagnosis codes [4]. However, this study did not present data for anxiety separately [4]. Previous research has also shown after the 2006 QOF was introduced, GP use of depression symptom codes increased [21]. Conversely, in the present study, there was no corresponding increase in anxiety symptom codes after the QOF, rather a reduction in the rate of increase in incidence occurred after 2007. The latter may reflect increasing presentation to IAPT (introduced in 2007/2008). However, this is unlikely as any changes in incidence would have been expected to have been seen over a prolonged period. Whilst there are no qualitative data from the previous studies [3–5, 21] to provide insight into possible reasons for the trends seen, previous interview data—based on vignettes—suggests GPs are reluctant to code for an anxiety disorder and prefer to use symptom codes where possible [8]. Our interview findings align with this.

However, a preference for using symptom codes does not explain why the present study found an increase in diagnostic codes in recent years. This may, in part, be due to the impact of the 2008 recession, as diagnosis codes levelled off between 2008 and 2013 after a previously sharp decline. Previous studies have also found reversals in declining suicide rates, and increasing rates of depression for several years after the recession [22, 23]. It may also be explained by the 2011 NICE anxiety guidelines, with the recommendation for earlier diagnosis increasing awareness among GPs [7]. Indeed, this study found an increase in the incidence rate of any anxiety code—symptom or diagnosis—after 2011. Finally, mental health campaigns may be increasing help-seeking behaviour. The anti-stigma campaign, ‘Time To Change’, led to an increase in intended help-seeking from GPs, and an increase in mental health discussion and disclosure [24]. This may also explain the rise in anxiety diagnoses (and symptoms) found in the later years of the present study.

The finding of an increased incidence of anxiety in females compared with males is consistent with previous research in primary care [2, 3]. Martin-Merino et al. [2] also found that between 2002 and 2004, the incidence of any anxiety code was highest in adults aged 20–29 years. However, the study did not distinguish between disorder codes and symptom codes and included a broader range of READ codes, including phobias. The present study has extended these findings and found that, in recent years, there has been a substantial increase in the recorded incidence of anxiety—diagnoses and symptoms—in young adults (aged < 35). This reflects national survey data, where 16–24-year-olds were nearly ten times more likely to report a mental health condition in 2014, compared with 1995 [25]. The authors suggest this may be attributed to social media, increased awareness and pressure on this generation. GPs interviewed in the present study also endorsed similar reasons [25].

This study predates the COVID-19 pandemic and some causes identified by GPs will have intensified, particularly social isolation [26]. However, the pandemic may have resulted in further increases in anxiety by its impact on various groups: those infected with COVID-19 and those experiencing longer-term health consequences (long COVID); those with increased health anxiety due to concerns about themselves and/or family/friends catching the virus; those experiencing financial difficulties due to the economic impact; and young adults whose education or career prospects have been affected [27–29]. This impact—at a time when the incidence of anxiety was already increasing—may have longer-term consequences for individuals and society.

### Implications

The incidence of recorded anxiety diagnoses decreased between 2003 and 2008, but increased from 2013 to 2018. In contrast, the incidence of recorded anxiety symptoms increased over the 16-years. The increase in the incidence of both diagnosis and symptom codes in recent years was particularly marked in young adults. GP interview data suggests the earlier decline in the recording of anxiety diagnoses may have been due to a preference for using symptom codes, rather than diagnosis codes. However, increased awareness of anxiety among patients may be increasing primary care presentation, and hence driving recent increases in recorded anxiety, especially in young adults. GPs suggested that pressure to succeed, and social media use, may be contributing to this.

There is a need for future research to understand the rise in anxiety seen in recent years—particularly amongst young adults. This may be critical in the development of interventions for the prevention and treatment of anxiety, and ultimately, lead to better outcomes for these patients.

## Supplementary Information

Below is the link to the electronic supplementary material.

Supplementary file1 (DOCX 1553 kb)

## Data Availability

The CPRD dataset reported in this work is not publicly available due to a lack of informed consent and ethical approval for open access, but can be requested from CPRD. Requests require a protocol describing the purpose of data access, ethical approval at the applicant’s institution, and provision for secure data access. Anonymised qualitative interview transcript data will be stored on the University of Bristol’s Research Data Service repository. Bonafide researchers will be able to access this data subject to a data access agreement and following approval from the University of Bristol Data Access Committee.

## Code availability

Stata code is available on request from the corresponding author.
