# Supplementary material for: Trends in the recording of anxiety in UK primary care: a multi-method approach
Source: Soc Psychiatry Psychiatr Epidemiol. 2021 Jul 1;57(2):375–86. doi: 10.1007/s00127-021-02131-8 (PMC8246441; doi:10.1007/s00127-021-02131-8)
Supplement: Supplementary file 1 — Supplementary file1 (DOCX 1553 kb) [file 127_2021_2131_MOESM1_ESM.docx]

**Social Psychiatry and Psychiatric Epidemiology**

**Trends in the recording of anxiety in UK primary care: a multi-method approach – supplementary information**

Charlotte Archer^1^, Katrina Turner^1,3^, David Kessler^1,2^, Becky Mars^1,2^ and Nicola Wiles^1,2^.

^1^Bristol Medical School, University of Bristol, UK

^2^NIHR Bristol Biomedical Research Centre, UK.

^3^The National Institute for Health Research, Applied Research Collaboration West (NIHR ARC West), University Hospitals Bristol NHS Foundation Trust, Bristol, UK.

Email for correspondence: [charlotte.archer@bristol.ac.uk](mailto:charlotte.archer@bristol.ac.uk)

1. Anxiety READ codes
2. Best-fitting join point model of incidence of any anxiety code per 1000PYAR
3. Best-fitting join point model of incidence of diagnosis codes per 1000PYAR
4. Best-fitting join point model of incidence of symptom codes per 1000PYAR
5. Incidence rate ratios for GP recorded anxiety – any anxiety code
6. Incidence rate ratios for GP recorded anxiety - diagnosis codes
7. Incidence rate ratios for GP recorded anxiety - symptom codes
8. Incidence of GP recorded anxiety (any anxiety code) per 1000 PYAR by gender
9. Incidence of GP recorded anxiety (diagnosis codes) per 1000 PYAR by gender
10. Incidence of GP recorded anxiety (symptom codes) per 1000 PYAR by gender
11. Incidence of GP recorded anxiety (diagnosis codes) per 1000 PYAR, by age
12. Incidence of GP recorded anxiety (symptom codes) per 1000 PYAR, by age
13. Incidence rates for GP recorded diagnosis codes - generalised anxiety (GAD), mixed anxiety and depression (MADD), and panic attack/disorder (Panic) - between 2003 and 2018

**Supplement 1 Anxiety READ codes**

1B13.11 Anxiousness symptom

1B12.11 Nerves

1B12.12 Tension - nervous

1B13.00 Anxiousness

1Bk.00 Worried

1B12.00 Nerves - nervousness

1B13.12 - Anxious

2258.00 O/E - anxious

225J.00 O/E panic attack

E200.00 Anxiety states [parent]

E200000 Anxiety state unspecified

E200100 Panic disorder

E200111 Panic attack

E200200 Generalised anxiety disorder

E200300 Anxiety with depression

E200400 Chronic anxiety

E200500 Recurrent anxiety

E200z00 Anxiety state NOS

E202100 Agoraphobia with panic attacks

E202.11 Social phobic disorders

E202200 Agoraphobia without mention of panic attacks

Eu34114 [X] Persistent anxiety depression

Eu40000 [X] Agoraphobia

Eu40011 [X] Agoraphobia without history of panic disorder

Eu40012 [X] Panic disorder with agoraphobia

Eu40100 [X] Social phobias

Eu40112 [X] Social neurosis

Eu41.00 [X] Other anxiety disorders

Eu41000 [X] Panic disorder [episodic paroxysmal anxiety]

Eu41011 [X] Panic attack

Eu41012 [X] Panic state

Eu41100 [X] Generalised anxiety disorders

Eu41111 [X] Anxiety neurosis

Eu41113 [X] Anxiety state

Eu41200 [X] Mixed anxiety and depressive disorder

Eu41211 [X] Mild anxiety depression

Eu41300 [X] Other mixed anxiety disorders

Eu41y00 [X] Other specified anxiety disorders

Eu41z00 [X] Anxiety disorder, unspecified

Eu41z11 [X] Anxiety NOS


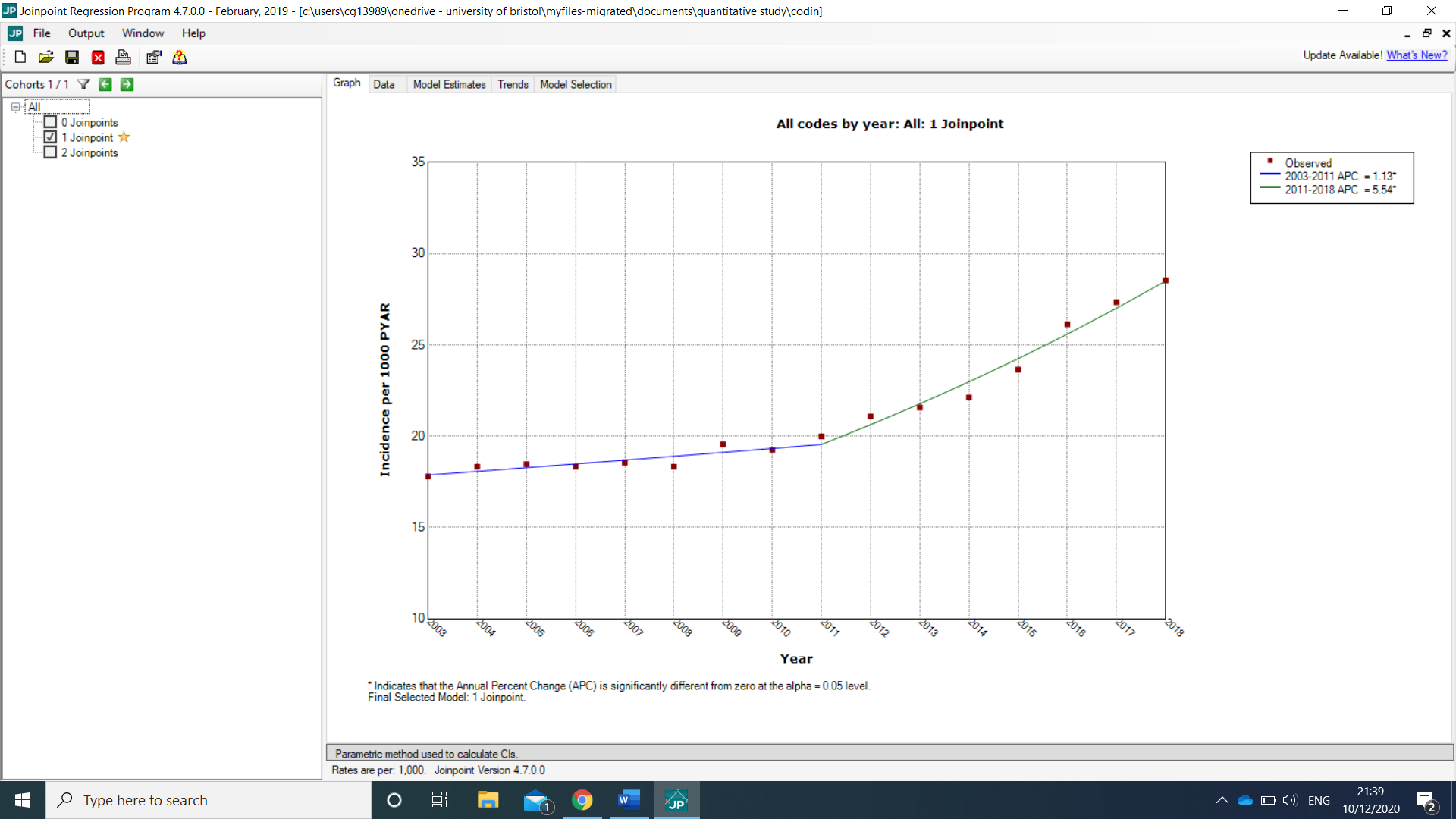


Supplement 2 Best-fitting join point model of incidence of any anxiety code per 1000PYAR

**The annual percentage change (APC) is based on the slope of each line ‘segment’ between joinpoints*


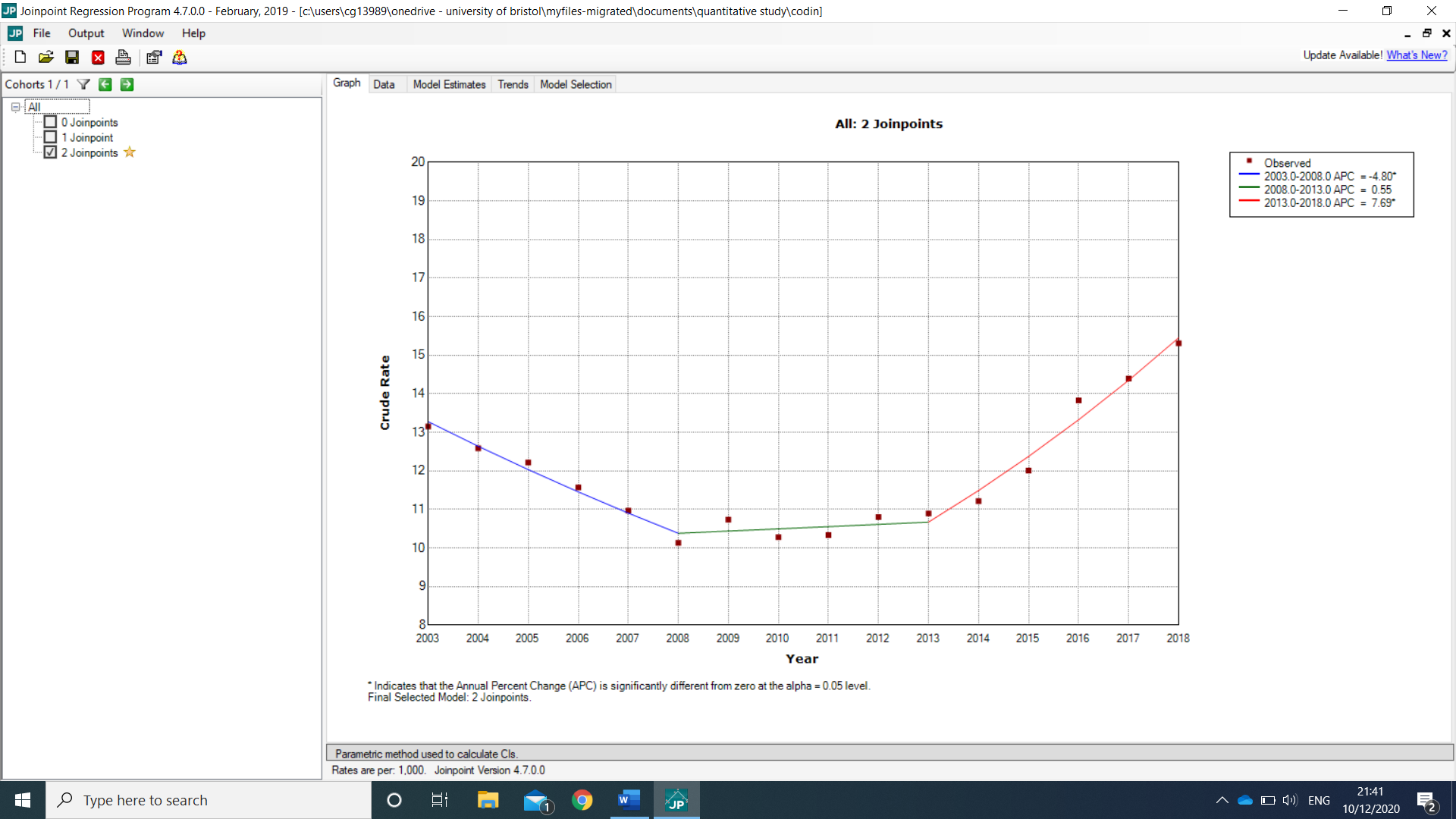


Supplement 3 Best-fitting join point model of incidence of diagnosis codes per 1000PYAR

**The annual percentage change (APC) is based on the slope of each line ‘segment’ between joinpoints*


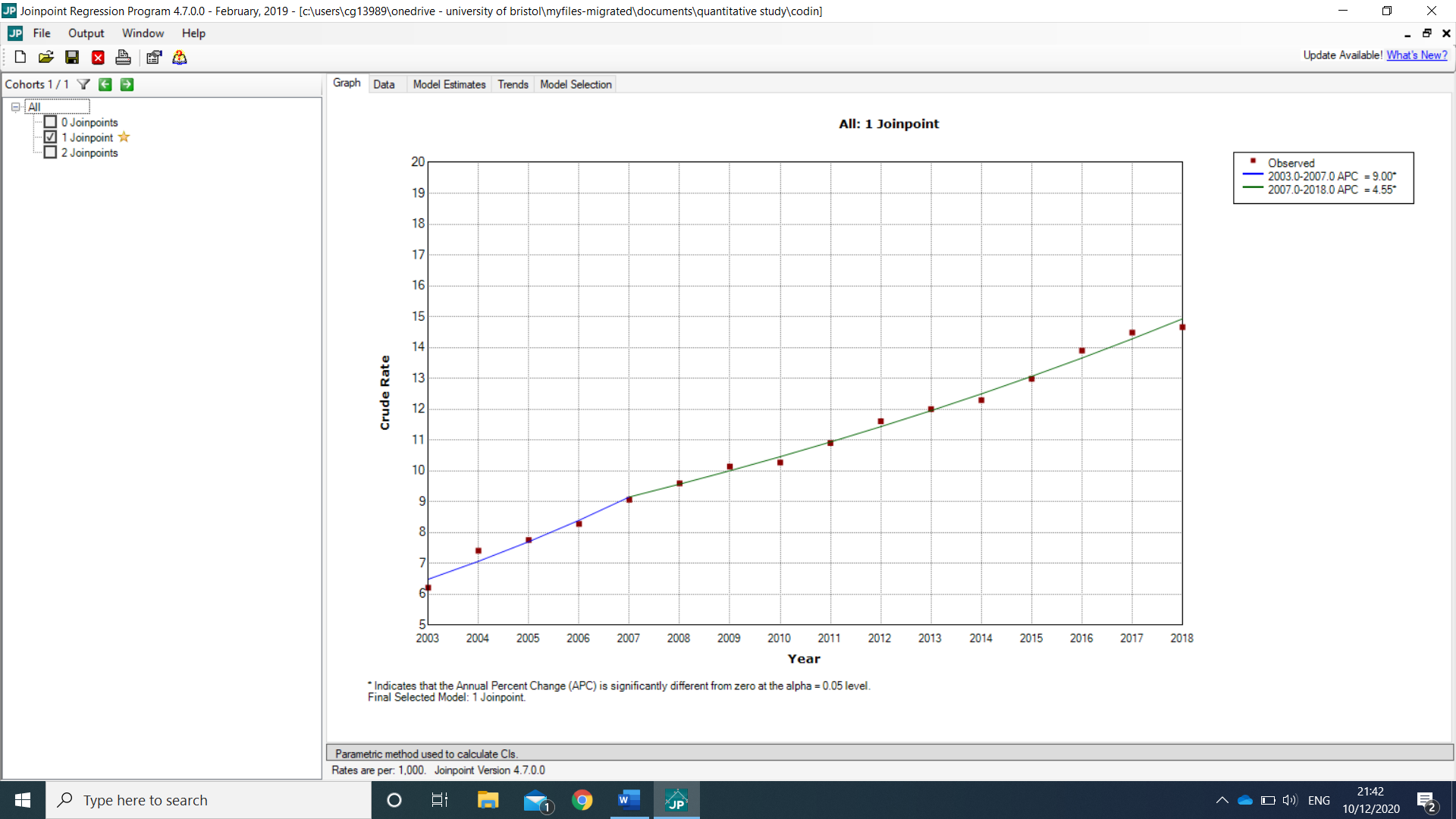


Supplement 4 Best-fitting join point model of incidence of symptom codes per 1000PYAR

**The annual percentage change (APC) is based on the slope of each line ‘segment’ between joinpoints*

Supplement 5 Incidence rate ratios for GP recorded anxiety – any anxiety code

| Variable | | Any anxiety code | | | | | |
| --- | --- | --- | --- | --- | --- | --- | --- |
|  |  | Univariable IRR | (95%CI) | P value | Multivariable IRR* | (95%CI) | P value |
| Year | 2003 | 1.00 |  | <0.001 | 1.00 |  | <0.001 |
|  | 2004 | 1.03 | (1.01-1.05) |  | 1.03 | (1.01-1.05) |  |
|  | 2005 | 1.04 | (1.02-1.06) |  | 1.04 | (1.02-1.07) |  |
|  | 2006 | 1.03 | (1.01-1.05) |  | 1.04 | (1.02-1.06) |  |
|  | 2007 | 1.04 | (1.02-1.06) |  | 1.05 | (1.03-1.07) |  |
|  | 2008 | 1.03 | (1.01-1.05) |  | 1.04 | (1.02-1.06) |  |
|  | 2009 | 1.10 | (1.08-1.12) |  | 1.12 | (1.09-1.14) |  |
|  | 2010 | 1.08 | (1.06-1.10) |  | 1.10 | (1.08-1.12) |  |
|  | 2011 | 1.12 | (1.10-1.15) |  | 1.14 | (1.12-1.17) |  |
|  | 2012 | 1.19 | (1.16-1.21) |  | 1.21 | (1.18-1.23) |  |
|  | 2013 | 1.21 | (1.19-1.24) |  | 1.24 | (1.21-1.26) |  |
|  | 2014 | 1.24 | (1.22-1.27) |  | 1.27 | (1.25-1.30) |  |
|  | 2015 | 1.33 | (1.31-1.35) |  | 1.36 | (1.34-1.39) |  |
|  | 2016 | 1.47 | (1.44-1.50) |  | 1.51 | (1.48-1.54) |  |
|  | 2017 | 1.54 | (1.51-1.56) |  | 1.58 | (1.55-1.61) |  |
|  | 2018 | 1.60 | (1.58-1.63) |  | 1.65 | (1.63-1.68) |  |
| Gender | Male | 1.00 |  | <0.001 | 1.00 |  | <0.001 |
|  | Female | 2.09 | (2.07-2.10) |  | 2.13 | (2.11-2.14) |  |
| Age Band  (years) | 18-24 | 1.00 |  | <0.001 | 1.00 |  | <0.001 |
|  | 25-34 | 1.09 | (1.08-1.10) |  | 1.08 | (1.07-1.10) |  |
|  | 35-44 | 1.03 | (1.02-1.05) |  | 1.05 | (1.04-1.06) |  |
|  | 44-54 | 0.97 | (0.96-0.98) |  | 0.97 | (0.96-0.98) |  |
|  | 55-64 | 0.84 | (0.83-0.85) |  | 0.84 | (0.83-0.85) |  |
|  | 65-74 | 0.74 | (0.73-0.75) |  | 0.72 | (0.71-0.73) |  |
|  | 75-84 | 0.77 | (0.76-0.79) |  | 0.73 | (0.72-0.74) |  |
|  | 85+ | 0.67 | (0.65-0.68) |  | 0.58 | (0.57-0.60) |  |
| **Multivariable model adjusted for year, gender, and age band* | | | | | | | |

Supplement 6 Incidence rate ratios for GP recorded anxiety - diagnosis codes

| Variable | | Diagnosis codes | | | | | |
| --- | --- | --- | --- | --- | --- | --- | --- |
|  |  | Univariable IRR | (95%CI) | P value | Multivariable IRR* | (95%CI) | P value |
| Year | 2003 | 1.00 |  | <0.001 | 1.00 |  | <0.001 |
|  | 2004 | 0.96 | (0.94-0.98) |  | 0.96 | (0.94-0.98) |  |
|  | 2005 | 0.93 | (0.91-0.95) |  | 0.93 | (0.91-0.96) |  |
|  | 2006 | 0.88 | (0.86-0.90) |  | 0.89 | (0.87-0.91) |  |
|  | 2007 | 0.83 | (0.81-0.85) |  | 0.84 | (0.82-0.86) |  |
|  | 2008 | 0.77 | (0.75-0.79) |  | 0.78 | (0.76-0.80) |  |
|  | 2009 | 0.82 | (0.80-0.84) |  | 0.83 | (0.81-0.85) |  |
|  | 2010 | 0.78 | (0.76-0.80) |  | 0.79 | (0.77-0.81) |  |
|  | 2011 | 0.79 | (0.77-0.81) |  | 0.80 | (0.78-0.82) |  |
|  | 2012 | 0.82 | (0.80-0.84) |  | 0.84 | (0.82-0.86) |  |
|  | 2013 | 0.83 | (0.81-0.85) |  | 0.84 | (0.82-0.86) |  |
|  | 2014 | 0.85 | (0.83-0.87) |  | 0.87 | (0.85-0.89) |  |
|  | 2015 | 0.91 | (0.89-0.93) |  | 0.93 | (0.91-0.96) |  |
|  | 2016 | 1.05 | (1.03-1.08) |  | 1.08 | (1.05-1.10) |  |
|  | 2017 | 1.09 | (1.07-1.12) |  | 1.12 | (1.10-1.15) |  |
|  | 2018 | 1.16 | (1.14-1.19) |  | 1.20 | (1.17-1.22) |  |
| Gender | Male | 1.00 |  | <0.001 | 1.00 |  | <0.001 |
|  | Female | 2.03 | (2.01-2.05) |  | 2.07 | (2.05-2.09) |  |
| Age Band  (years) | 18-24 | 1.00 |  | <0.001 | 1.00 |  | <0.001 |
|  | 25-34 | 1.09 | (1.07-1.11) |  | 1.08 | (1.06-1.10) |  |
|  | 35-44 | 1.06 | (1.04-1.07) |  | 1.06 | (1.04-1.08) |  |
|  | 44-54 | 0.96 | (0.94-0.97) |  | 0.96 | (0.94-0.97) |  |
|  | 55-64 | 0.81 | (0.79-0.82) |  | 0.80 | (0.79-0.81) |  |
|  | 65-74 | 0.65 | (0.64-0.67) |  | 0.64 | (0.62-0.65) |  |
|  | 75-84 | 0.66 | (0.64-0.67) |  | 0.62 | (0.61-0.63) |  |
|  | 85+ | 0.54 | (0.53-0.56) |  | 0.48 | (0.46-0.50) |  |
| **Multivariable model adjusted for year, gender, and age band* | | | | | | | |

Supplement 7 Incidence rate ratios for GP recorded anxiety - symptom codes

| Variable | | Symptom codes | | | | | |
| --- | --- | --- | --- | --- | --- | --- | --- |
|  |  | Univariable IRR | (95%CI) | P value | Multivariable IRR | (95%CI) | P value |
| Year | 2003 | 1.00 |  | <0.001 | 1.00 |  | <0.001 |
|  | 2004 | 1.19 | (1.16-1.23) |  | 1.20 | (1.16-1.23) |  |
|  | 2005 | 1.25 | (1.21-1.29) |  | 1.25 | (1.21-1.29) |  |
|  | 2006 | 1.33 | (1.29-1.37) |  | 1.34 | (1.30-1.38) |  |
|  | 2007 | 1.46 | (1.42-1.50) |  | 1.47 | (1.43-1.52) |  |
|  | 2008 | 1.54 | (1.50-1.59) |  | 1.56 | (1.51-1.60) |  |
|  | 2009 | 1.63 | (1.58-1.68) |  | 1.65 | (1.60-1.70) |  |
|  | 2010 | 1.65 | (1.60-1.70) |  | 1.67 | (1.62-1.72) |  |
|  | 2011 | 1.75 | (1.70-1.81) |  | 1.77 | (1.72-1.83) |  |
|  | 2012 | 1.87 | (1.82-1.92) |  | 1.89 | (1.84-1.95) |  |
|  | 2013 | 1.93 | (1.88-1.99) |  | 1.96 | (1.90-2.01) |  |
|  | 2014 | 1.98 | (1.92-2.04) |  | 2.01 | (1.95-2.07) |  |
|  | 2015 | 2.09 | (2.03-2.15) |  | 2.12 | (2.06-2.19) |  |
|  | 2016 | 2.24 | (2.17-2.30) |  | 2.28 | (2.21-2.34) |  |
|  | 2017 | 2.33 | (2.27-2.40) |  | 2.38 | (2.31-2.44) |  |
|  | 2018 | 2.36 | (2.29-2.43) |  | 2.41 | (2.34-2.48) |  |
| Gender | Male | 1.00 |  | <0.001 | 1.00 |  | <0.001 |
|  | Female | 2.09 | (2.07-2.11) |  | 2.12 | (2.10-2.14) |  |
| Age Band  (years) | 18-24 | 1.00 |  | <0.001 | 1.00 |  | <0.001 |
|  | 25-34 | 1.08 | (1.06-1.10) |  | 1.07 | (1.05-1.09) |  |
|  | 35-44 | 0.98 | (0.96-0.99) |  | 1.00 | (0.98-1.01) |  |
|  | 44-54 | 0.94 | (0.92-0.95) |  | 0.93 | (0.91-0.95) |  |
|  | 55-64 | 0.84 | (0.82-0.85) |  | 0.83 | (0.82-0.85) |  |
|  | 65-74 | 0.81 | (0.79-0.83) |  | 0.78 | (0.77-0.80) |  |
|  | 75-84 | 0.88 | (0.86-0.90) |  | 0.83 | (0.82-0.85) |  |
|  | 85+ | 0.77 | (0.75-0.80) |  | 0.67 | (0.65-0.69) |  |
| **Multivariable model adjusted for year, gender, and age band* | | | | | | | |

Supplement 8 Incidence of GP recorded anxiety (any anxiety code) per 1000 PYAR by gender


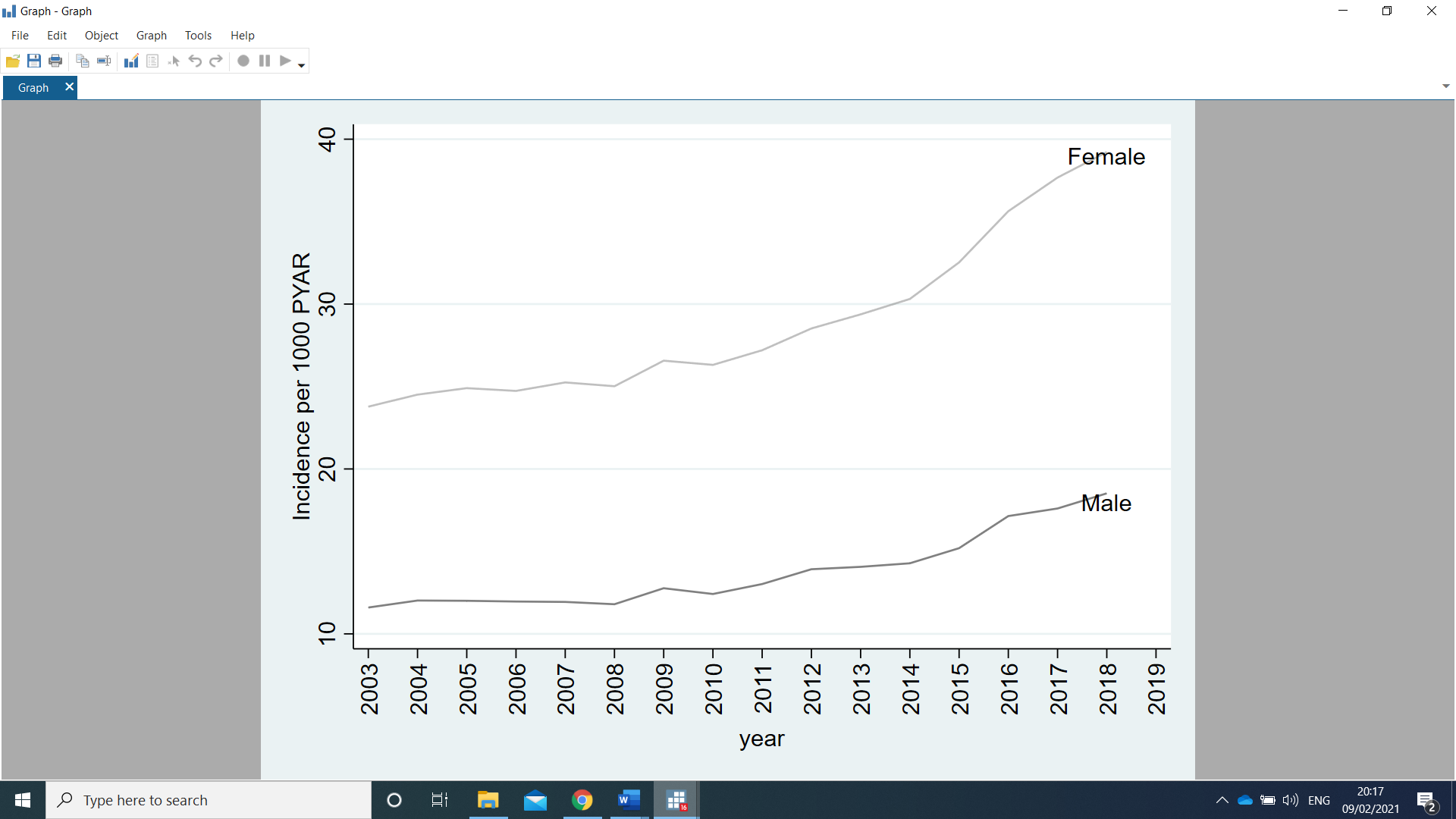

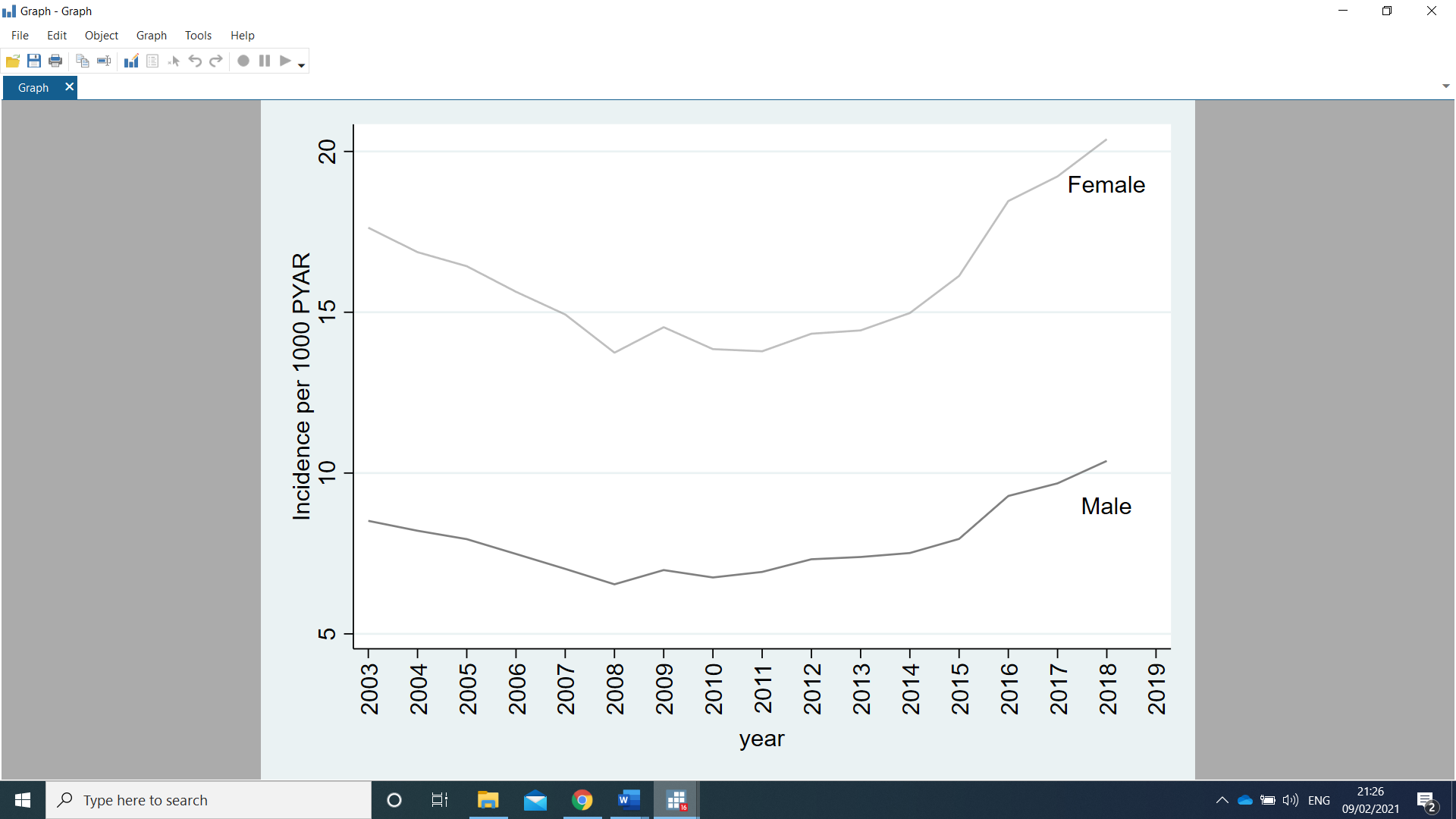


Supplement 9 Incidence of GP recorded anxiety (diagnosis codes) per 1000 PYAR by gender


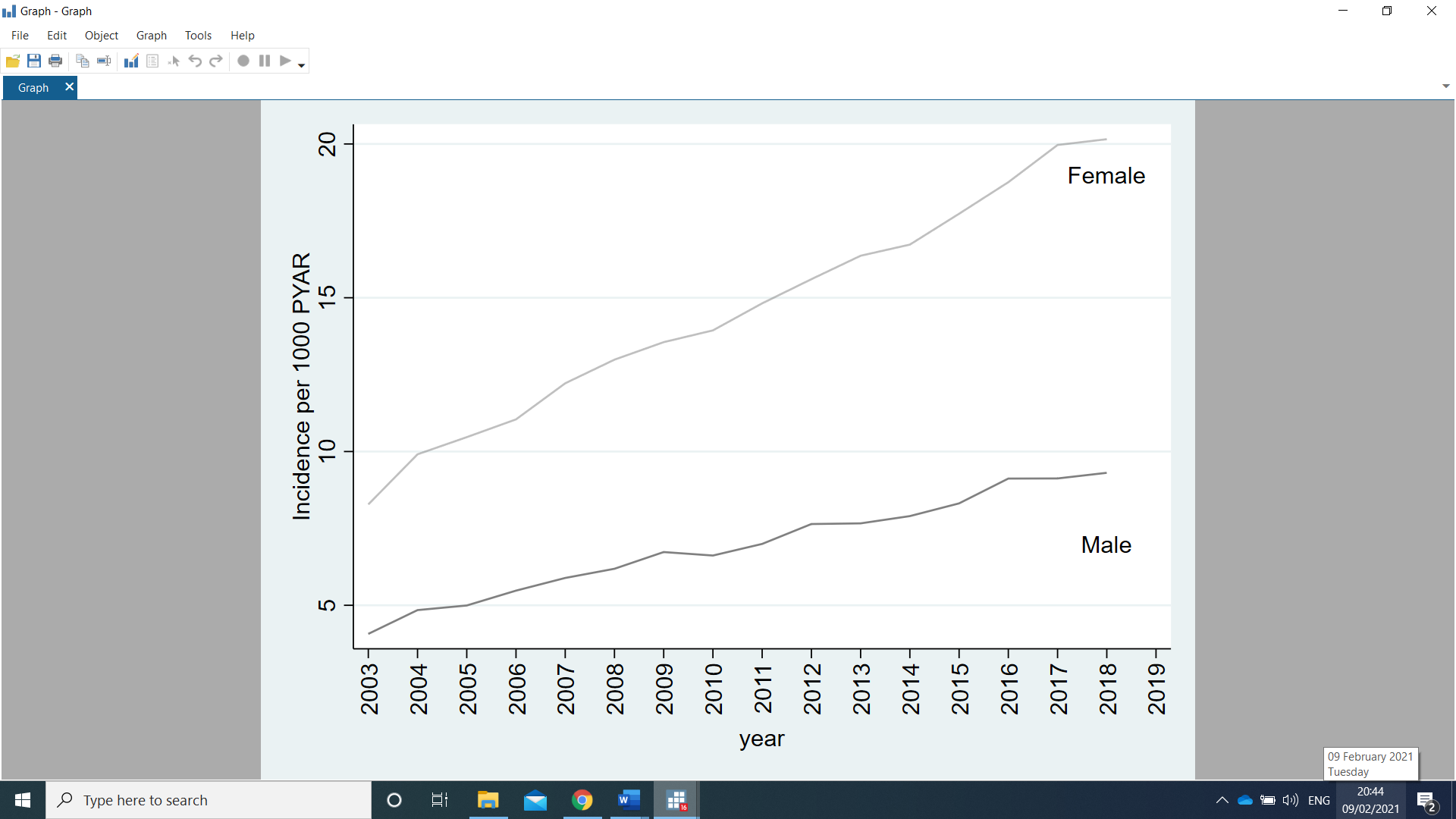


Supplement 10 Incidence of GP recorded anxiety (symptom codes) per 1000 PYAR by gender

Supplement 11 Incidence of GP recorded anxiety (diagnosis codes) per 1000 PYAR, by age


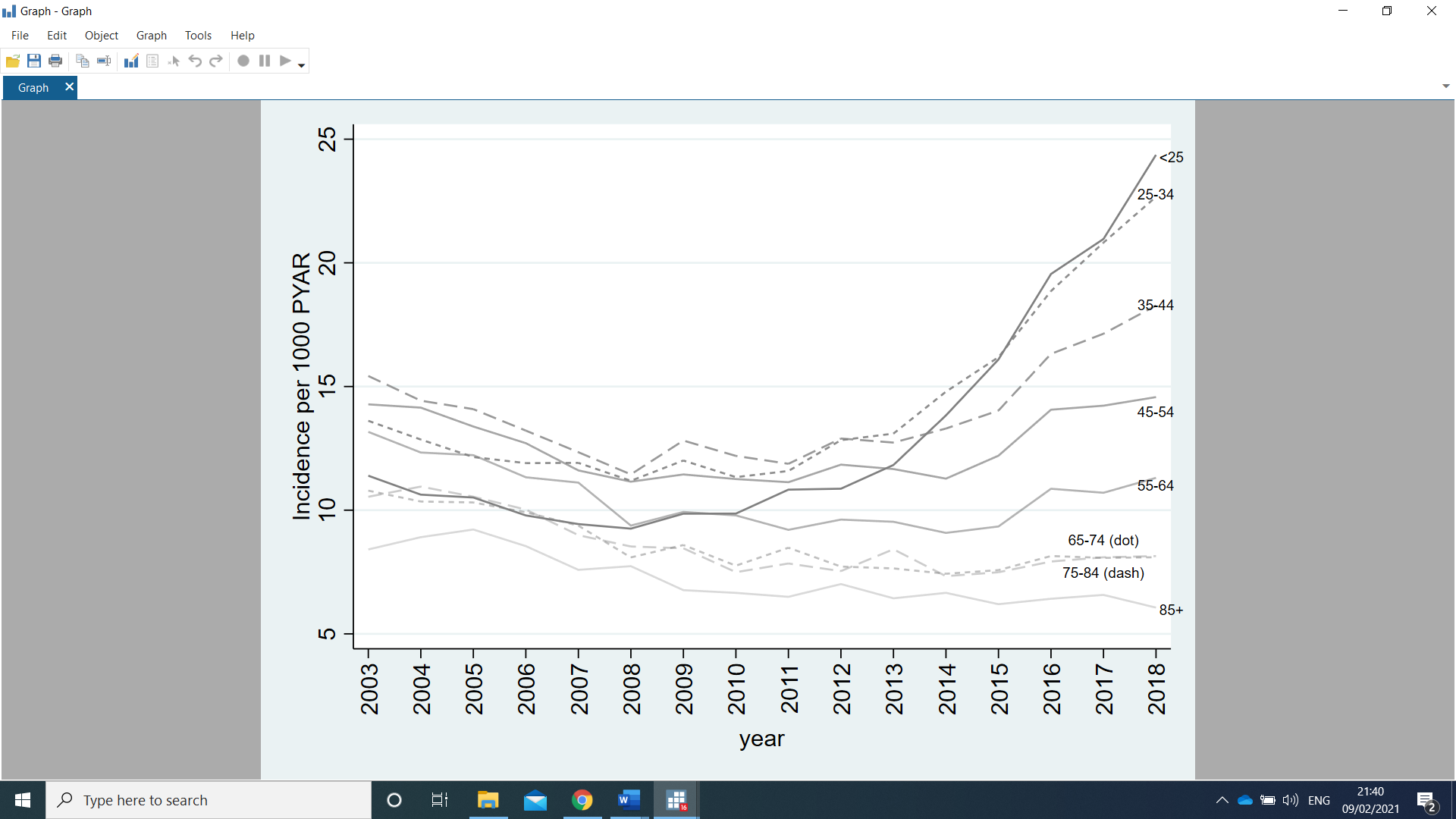


Supplement 12 Incidence of GP recorded anxiety (symptom codes) per 1000 PYAR, by age


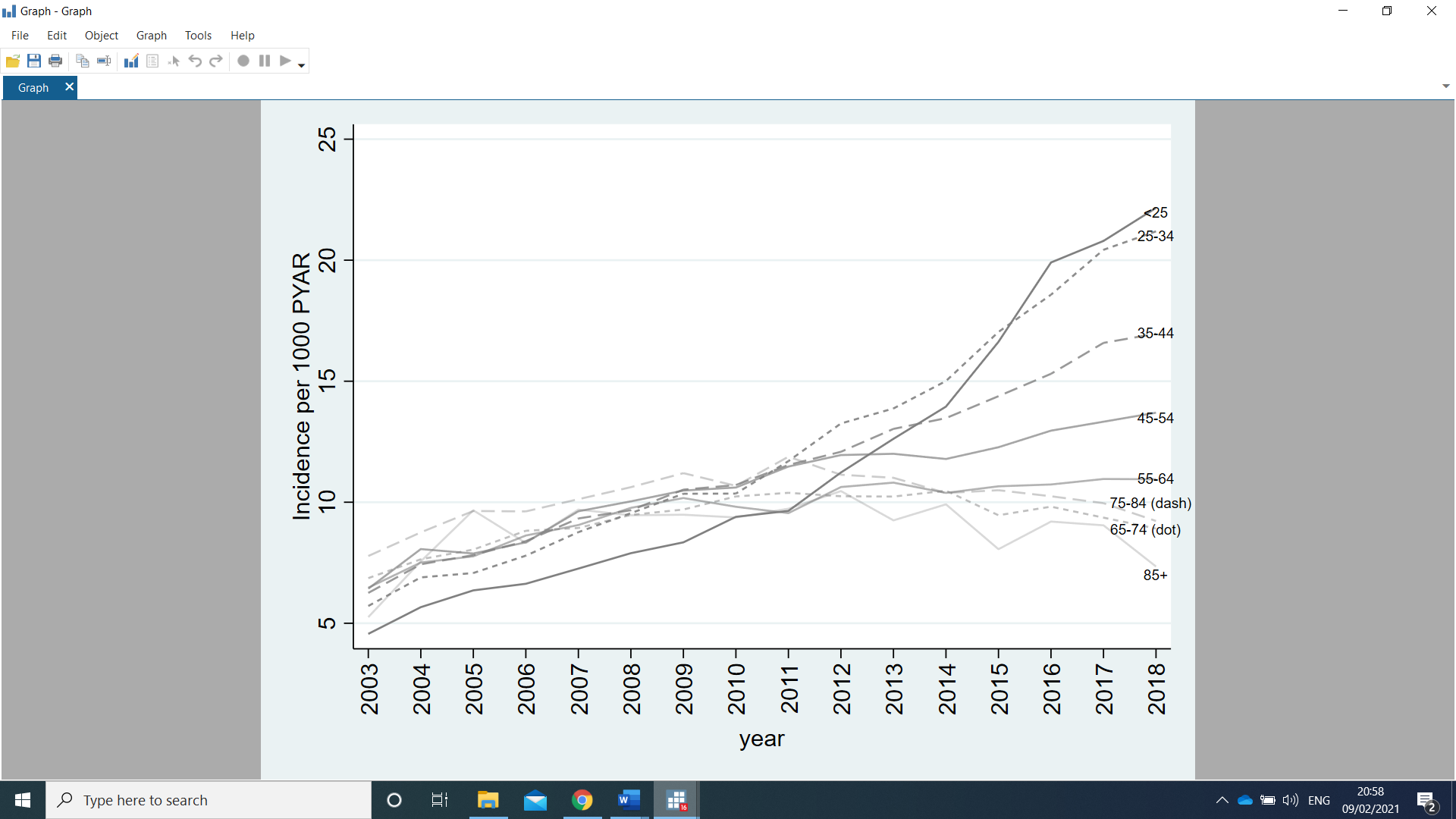


Supplement 13 Incidence rates for GP recorded diagnosis codes - generalised anxiety (GAD), mixed anxiety and depression (MADD), and panic attack/disorder (Panic) - between 2003 and 2018

| Variable | | GAD codes | | | | MADD codes | | | | Panic codes | | | | |
| --- | --- | --- | --- | --- | --- | --- | --- | --- | --- | --- | --- | --- | --- | --- |
|  |  | N of events | PYAR | Incidence (1000PYAR) | (95%CI) | N of events | PYAR | Incidence (1000PYAR) | (95%CI) | N of events | PYAR | Incidence (1000PYAR) | (95%CI) |  |
| Year | 2003 | 7735 | 1110795 | 7.0 | (6.8-7.1) | 5345 | 1111906 | 4.8 | (4.7-5.0) | 2713 | 1113259 | 2.4 | (2.4-2.5) |  |
|  | 2004 | 7366 | 1118504 | 6.6 | (6.4-6.7) | 4914 | 1121800 | 4.4 | (4.3-4.5) | 2821 | 1125367 | 2.5 | (2.4-2.6) |  |
|  | 2005 | 7175 | 1117956 | 6.4 | (6.3-6.9) | 4676 | 1123313 | 4.2 | (4.0-4.3) | 2708 | 1128498 | 2.4 | (2.3-2.5) |  |
|  | 2006 | 6915 | 1125687 | 6.1 | (6.0-6.3) | 4209 | 1132822 | 3.7 | (3.6-3.8) | 2626 | 1139307 | 2.3 | (2.2-2.4) |  |
|  | 2007 | 6449 | 1131423 | 5.7 | (5.6-5.8) | 4015 | 1140100 | 3.5 | (3.4-3.6) | 2473 | 1147528 | 2.2 | (2.1-2.2) |  |
|  | 2008 | 6078 | 1142584 | 5.3 | (5.1-5.5) | 3547 | 1152729 | 3.1 | (3.0-3.2) | 2280 | 1160912 | 2.0 | (1.9-2.1) |  |
|  | 2009 | 6712 | 1147923 | 5.9 | (5.7-6.0) | 3548 | 1159565 | 3.1 | (3.0-3.2) | 2407 | 1168332 | 2.1 | (2.0-2.1) |  |
|  | 2010 | 6392 | 1156026 | 5.5 | (5.4-5.7) | 3586 | 1169326 | 3.1 | (3.0-3.2) | 2132 | 1178626 | 1.8 | (1.7-1.9) |  |
|  | 2011 | 6763 | 1161722 | 5.8 | (5.7-6.0) | 3450 | 1176368 | 2.9 | (2.8-3.0) | 1980 | 1186411 | 1.7 | (1.6-1.7) |  |
|  | 2012 | 6966 | 1173032 | 5.9 | (5.8-6.1) | 3780 | 1189160 | 3.2 | (3.1-3.3) | 2126 | 1199951 | 1.8 | (1.7-1.9) |  |
|  | 2013 | 7540 | 1177990 | 6.4 | (6.3-6.6) | 3671 | 1195544 | 3.1 | (3.0-3.2) | 1876 | 1207214 | 1.6 | (1.5-1.6) |  |
|  | 2014 | 7899 | 1186763 | 6.7 | (6.5-6.8) | 3930 | 1206211 | 3.3 | (3.2-3.4) | 1803 | 1218763 | 1.5 | (1.4-1.6) |  |
|  | 2015 | 8374 | 1195608 | 7.0 | (6.9-7.2) | 4652 | 1216622 | 3.8 | (3.7-3.9) | 1550 | 1230583 | 1.3 | (1.2-1.3) |  |
|  | 2016 | 9558 | 1206451 | 7.9 | (7.8-8.1) | 6021 | 1228668 | 4.9 | (4.8-5.0) | 1502 | 1245057 | 1.2 | (1.2-1.3) |  |
|  | 2017 | 9884 | 1211024 | 8.2 | (8.0-8.3) | 6587 | 1234188 | 5.3 | (5.2-5.5) | 1431 | 1253591 | 1.1 | (1.1-1.2) |  |
|  | 2018 | 10021 | 1217166 | 8.2 | (8.1-8.4) | 7719 | 1240802 | 6.2 | (6.1-6.4) | 1271 | 1263629 | 1.0 | (1.0-1.1) |  |
